# The macrophage activation marker soluble CD163 is elevated and associated with liver disease phenotype in patients with Wilson’s disease

**DOI:** 10.1186/s13023-020-01452-2

**Published:** 2020-07-02

**Authors:** Emilie Glavind, Daniel N. Gotthardt, Jan Pfeiffenberger, Thomas Damgaard Sandahl, Teodora Bashlekova, Gro Linno Willemoe, Jane Preuss Hasselby, Karl Heinz Weiss, Holger Jon Møller, Hendrik Vilstrup, William M. Lee, Michael L. Schilsky, Peter Ott, Henning Grønbæk

**Affiliations:** 1 Department of Hepatology and Gastroenterology, Aarhus University Hospital, 99 Palle Juul-Jensens Boulevard, DK-8200 Aarhus N, Denmark; 2 Department of Internal Medicine IV, University Hospital Heidelberg, Heidelberg, Germany; 3 Department of Pathology, Copenhagen University Hospital, Rigshospitalet, Copenhagen, Denmark; 4 Department of Clinical Biochemistry, Aarhus University Hospital, Aarhus, Denmark; 5 Division of Digestive and Liver Diseases, UT Southwestern Medical Center at Dallas, Dallas, TX USA; 6 Yale University Medical Center, New Haven, CT 06520 USA

**Keywords:** Wilson’s disease, Macrophage activation, Liver cirrhosis, Acute liver failure, Biomarker

## Abstract

**Background:**

Macrophages play a significant role in liver disease development and progression. The macrophage activation marker soluble (s)CD163 is associated with severity and prognosis in a number of different acute and chronic liver diseases but has been only sparsely examined in Wilson’s disease (WD). We investigated sCD163 levels in patients with acute and chronic WD and hypothesized associations with liver disease phenotype and biochemical markers of liver injury.

**Methods:**

We investigated sCD163 in two independent cohorts of WD patients: 28 patients with fulminant WD from the US Acute Liver Failure (ALF) Study Group registry and 147 patients with chronic disease from a German WD registry. We included a control group of 19 healthy individuals. Serum sCD163 levels were measured by ELISA. Liver CD163 expression was determined by immunohistochemistry.

**Results:**

In the ALF cohort, median sCD163 was 10-fold higher than in healthy controls (14.6(2.5–30.9) vs. 1.5(1.0–2.7) mg/L, *p* < 0.001). In the chronic cohort, median sCD163 was 2.6(0.9–24.9) mg/L. There was no difference in sCD163 according to subgroups based on initial clinical presentation, i.e. asymptomatic, neurologic, hepatic, or mixed. Patients with cirrhosis at the time of diagnosis had higher sCD163 compared with those without cirrhosis (3.0(1.2–24.9) vs. 2.3(0.9–8.0) mg/L, *p* < 0.001); and both cohorts significantly lower than the ALF patients. Further, sCD163 correlated positively with ALT, AST, GGT and INR (rho = 0.27–0.53); and negatively with albumin (rho = − 0.37), (*p* ≤ 0.001, all). We observed immunohistochemical CD163 expression in liver tissue from ALF patients.

**Conclusions:**

Although sCD163 is not specific for WD, it was elevated in WD patients, especially in those with ALF. Further, sCD163 was higher in patients with cirrhosis compared to patients without cirrhosis and associated with biochemical markers of liver injury and hepatocellular function. Thus, macrophage activation is evident in WD and associates with liver disease phenotype and biochemical parameters of liver disease. Our findings suggest that sCD163 may be used as a marker of liver disease severity in WD patients.

## Background

Wilson’s disease (WD) is an autosomal recessive inherited disorder of copper metabolism resulting from mutations in the *ATP7B* gene. The gene encodes the ATP7B protein that mediates the build-in of copper in ceruloplasmin and/or excretion of excess copper into the bile. Dysfunction of ATP7B causes copper accumulation in the body, particularly in the liver and brain [1]. The clinical presentation of WD can vary widely and the most common clinical presentations are neuropsychiatric or hepatic disease either alone or mixed while asymptomatic patients are typically detected by family screening. The manifestations of liver disease range from an asymptomatic state with only abnormal liver function tests to cirrhosis and even life-threatening acute liver failure. WD is fatal if left untreated [2–4]. While most treatments arrest the development of neurological symptoms, slow progression of liver disease to cirrhosis is not uncommon [5]. Liver function tests like alanine aminotransferase (ALT) and aspartate aminotransferase (AST) are used to monitor progression of liver disease [3], but seems to be of questionable value [6–8]. In this situation a reliable biomarker for progression of liver disease is needed.

The pathogenesis of hepatocyte injury in WD is still incompletely understood [9]. The liver pathology of WD is highly variable and range from steatosis, glycogenated nuclei in hepatocytes and focal hepatocellular necrosis to chronic hepatitis, and cirrhosis [10]. With progression of disease, mononuclear inflammatory infiltrates develop and Kupffer cell hyperplasia and macrophage activation is present, especially in advanced cases with fibrosis and cirrhosis [10]. Macrophages may be activated by diverse stimuli, e.g., via pathogen-associated molecular patterns (PAMPs) or damage-associated molecular patterns (DAMPs) from injured hepatocytes [11]. To which extend the activation of macrophages is involved in the pathogenesis of WD remains unknown.

In a number of other liver diseases, activation of macrophages plays an important role in the development of liver inflammation, fibrosis and portal hypertension [12–14]. CD163, the hemoglobin-haptoglobin scavenger receptor, is lineage specific and expressed on the cell surface of macrophages and to some extent on monocytes. Upon macrophage activation, CD163 is shed and can be detected in the blood as soluble (s)CD163 [15] and used as a circulating biomarker of macrophage activation. We have previously shown that sCD163 is elevated in patients with a variety of inflammatory liver diseases with increasing levels dependent on liver disease severity from non-alcoholic fatty liver disease [16], chronic viral hepatitis [17], autoimmune hepatitis [18] and alcoholic hepatitis [19]. We also demonstrated clear associations between sCD163, liver disease severity (Child-Pugh and MELD scores), and portal hypertension in patients with cirrhosis [20]. Furthermore, sCD163 increased in a stepwise manner in patients with increasing grades of acute-on-chronic liver failure [21]; however, the highest levels are observed in patients with acute liver failure (ALF) [22].

A pilot study from our laboratory suggested that sCD163 was elevated in WD patients and may be a biomarker of progression of liver disease [23]. In the present study, we aimed to investigate macrophage activation by sCD163 serum levels in a larger group of patients with WD and different liver disease phenotypes ranging from chronic liver disease to ALF.

## Materials and methods

### Patients

We investigated 28 patients with WD from the US Acute Liver Failure (ALF) Study Group registry and 147 patients with WD from a German WD registry including patients with chronic WD in a retrospective cohort study. We used a control group of 19 healthy individuals [19].

In the US ALF cohort, 24 patients met ALF criteria of coagulopathy (International normalized ratio (INR) > 1.5) and any degree of hepatic encephalopathy and four patients presented with severe acute liver injury (ALI). It is understood that although fulminant WD presents as a newly diagnosed condition with rapid onset liver failure most if not all such patients have underlying cirrhosis. After informed consent was obtained from next of kin, detailed prospective clinical and laboratory data were entered in anonymous fashion into case report forms at admission to study and 3 weeks later, or at time of death or liver transplantation. Blood samples for sCD163 analyses were collected at day 1–7 and information on 21-day mortality was recorded. The study met all requirements of Institutional Review Boards at the respective study sites.

In the German cohort of 147 chronic patients the diagnosis of WD was established according to standard criteria [5, 24]. The *ATP7B* mutational analysis was performed as previously described [25, 26] in 100 of the 147 WD patients. Data on initial presentation were recorded and patients categorized into subgroups on the basis of symptoms present at the time of diagnosis: asymptomatic, neurologic, hepatic, or mixed presentation. No patient presented with ALF. The presence of Kayser–Fleischer rings was recorded. Cirrhosis was diagnosed by histology or on the presence of typical findings in imaging in combination with clinical signs of portal hypertension (splenomegaly, ascites or esophageal varices). Thirty patients (20%) were diagnosed by family screening. Blood samples for sCD163 determination and standard biochemical analyses were collected between January 2011 and June 2016, which was median 15 years (range = 0–50 years) after establishment of the WD diagnosis. Two patients (1%) were treatment naive and 145 patients (99%) were prescribed medicine for WD at the time of blood sample collection. The majority of patients were treated with a chelating agent, i.e. penicillamine or trientine (*n* = 116; 80%), and the minority with either zinc (*n* = 14; 10%) or a combination of a chelating agent and zinc (n = 11; 8%). In a few patients the type of medication was unknown (*n* = 4; 3%). Current medical treatment was also first-line treatment in 66 patients (46%). The median time under current medical treatment prior to blood sampling was 76 months (range = 0–547 months). Twenty-two patients were in the early phase of treatment, i.e. less than or equal to 12 months (median 3 months), and 123 patients beyond 12 months of treatment (median 104 months). All patients signed informed consent before participation in the study in accordance with the Helsinki Declaration, and the local Ethics Committee approved the study.

The historical healthy control group consisted of 9 females and 10 males with a median age of 44 years (range = 31–64 years).

### Biochemical analyses

The serum sCD163 levels were determined in duplicates in samples that had been frozen at − 80 °C by an in-house sandwich enzyme-linked immunosorbent assays (ELISA) using a BEP-2000 ELISA analyser (Dade Behring, Deerfield, IL, USA), as previously described [15]. Using the same assay, we have previously measured sCD163 in a large cohort (*n* = 240) of healthy subjects (median = 1.7 mg/L, reference interval = 0.7–3.9 mg/L) [27].

### Immunohistochemistry

Formalin-fixed and paraffin-embedded tissue from explanted liver tissue and core needle liver biopsies from 8 randomly selected WD patients from the US ALF cohort were obtained, and 4 μm sections were cut and mounted onto slides. All slides were stained with a CD163 antibody (clone MRQ-26 from Ventana Medical System Inc., Tucson, Arizona USA) as a ready-to-use formula with a dilution of 0,23 μl/ml. Pre-treatment was performed including incubation of the unstained slides for 4 min at temp. 72 °C, following rinse with EZ Prep (Ventana Medical Systems) and incubation with Cell Conditioner for 16–22 min. Incubation with the primary antibody was performed at temp. 36 °C for 24 min. Automated staining with Bench Mark Ultra nr. 233,378, Ventana Medical System Inc., Tucson, Arizona USA was applied. Nuclear counterstaining with Mayer’s hematoxylin was performed and the slides were coverslipped using the Tissue-Tek Prisma & Tissue-Tek Film (Sakura Finetek Europe B.V., Alphen aan den Rijn, The Netherlands). Positive controls included liver tissue, appendix and tonsil.

Semiquantitative evaluation was performed with an estimation of the overall expression of CD163 cells throughout the entire slide applying a scale from 0 to 3. The value 0 indicated no CD163 positive cells in the slide, 1 low density of CD163 positive cells with only very few cell-cell contacts, 2 moderate density of CD163 positive cells with more frequent cell-cell contacts, and 3 indicating high density of CD163 positive cells with many cell-cell contacts. Please see Supplementary Information for histology pictures illustrating the scoring algorithm. The samples were scored individually by two trained liver pathologists, and in cases of disagreement the samples were reviewed under a double-headed microscope and consensus was obtained.

Digital evaluation was performed applying an in-house customized CD163 targeted application on scanned slides. The slides were scanned using a Nano Zoomer slide scanner (Hamamatsu Photonics K.K., Japan) using 40 times resolution. The application designed, using the VIS program from Visiopharm (Visiopharm, Hørsholm, Denmark), was customized to estimate the area fraction (%) of CD163 positivity in the entire scanned slide.

### Statistical analysis

Differences between groups were assessed by unpaired t-tests (parametric, continuous variables) or Wilcoxon rank-sum tests (non-parametric, continuous variables). The assumption of normality was checked using quantile-quantile plots and histograms and we used logarithmic transformation to ensure a normal distribution where appropriate. The Kruskal-Wallis test was used for more than two groups, and significant results were further analysed. Changes within each group were assessed by Wilcoxon singed-rank test. Fisher’s exact test was used to assess differences in proportions. The Spearman rank-order correlation coefficient (rho) was used to estimate the associations between sCD163 and biochemical markers of liver injury, hepatocellular function and WD specific markers. We performed multiple logistic regression analysis to investigate the relationship between sCD163 and cirrhosis adjusting for demographic and biochemical parameters associated with cirrhosis and disease severity (age, gender, ALT, bilirubin, albumin, INR, creatinine). All continuous variables were logarithmically transformed. To identify candidate variables for a new CD163-based cirrhosis score we performed backward elimination based on the multiple logistic regression analysis with a significance limit of 0.1 with inclusion of significant parameters in the score. Non-parametric receiver-operating characteristics (ROC) analysis was used to assess the performance of sCD163 and of the CD163-based cirrhosis score in the prediction of liver cirrhosis and the two were compared using the test of equality of ROC areas. The Youden index method was used to determine the optimal cut-off value. Data are expressed as medians (ranges) for continuous variables and total number (%) for categorical variables except when otherwise specified. *P*-values ≤0.05 were considered statistically significant. Statistical analysis was performed using Stata 12.1 software from Stata Corporation (College Station, TX, USA).

## Results

### Patient characteristics

Demographic and biochemical data for both patient cohorts are provided in Table 1. On admission in the US ALF cohort, 17 patients had hepatic coma grade I or II, and 11 patients had grade III or IV hepatic encephalopathy; maximum hepatic encephalopathy was I or II in 8 patients, whereas 20 patients had maximum grade III or IV hepatic encephalopathy. In the German chronic cohort, 18 patients (12%) were asymptomatic, 20 patients (14%) had neurologic presentation, 77 patients (52%) hepatic, and 32 patients (22%) mixed hepatic and neurologic. Cirrhosis was diagnosed in 3/18 of the asymptomatic, 1/20 of the neurologic, 23/77 of the hepatic, and 13/32 of the patients with mixed presentation. Thus, in total, 40 patients (27%) had cirrhosis at diagnosis. Seventy patients (48%) presented with Kayser–Fleischer ring/rings.

**Table 1:** Patient characteristics

	German chronic cohort (***n*** = 147)	US ALF cohort (***n*** = 28)
Age at blood sample collection (years)	35 (16–69)	26 (15–57)
Female gender	79 (54%)	20 (71%)
ALT (U/L)	36 (10–436)	36 (7–1463)
AST (U/L)	30 (8–580)	188 (43–4481)
GGT (U/L)	33 (5–471)	–
Bilirubin (mg/dL)	0.7 (0.3–7.8)	31.4 (4.5–67.6)
Albumin (g/L)	44 (22–53)	24 (16–28)
Urea (kU/L)	6.1 (1.0–11.9)	–
Sodium (mmol/L)	–	139 (131–149)
Creatinine (mg/dL)	0.7 (0.4–2.4)	1.2 (0.4–7.1)
WBC count (× 10^9^/L)	5.9 (2.5–12.8)	13.4 (3.4–54.9)
INR	1.1 (0.9–2.0)	2.8 (1.7–6.5)
Phosphate (mmol/dL)	–	2.9 (1.0–11.7)
Serum copper (μmol/L)^†^	4.5 (0.1–29.9)	0.3 (0.2–208.0)
Ceruloplasmin (g/L)	0.10 (0.01–0.32)	0.17 (0.04–0.45)
Urinary copper (μmol/day)^†, ‡^	2.5 (0.2–27.5)	40,110.2 (661.4–9,931,842.2)

*ALT* alanine aminotransferase, *AST* aspartate aminotransferase, *GGT* gamma-glutamyltransferase, *WBC* white blood cell, *INR* international normalized ratio

Values are obtained at the same time as the blood samples used to measure soluble CD163

Data are medians (ranges) for continuous variables and total number (%) for categorical variables

^†^*n* = 17 in US ALF cohort

^‡^*n* = 108 in German chronic cohort

### CD163 in healthy controls

The median (range) plasma sCD163 in the 19 healthy controls was 1.5 (1.0–2.7) mg/L and similar to the normal values of our laboratory.

### CD163 in ALF WD patients

**Fig. 1:**
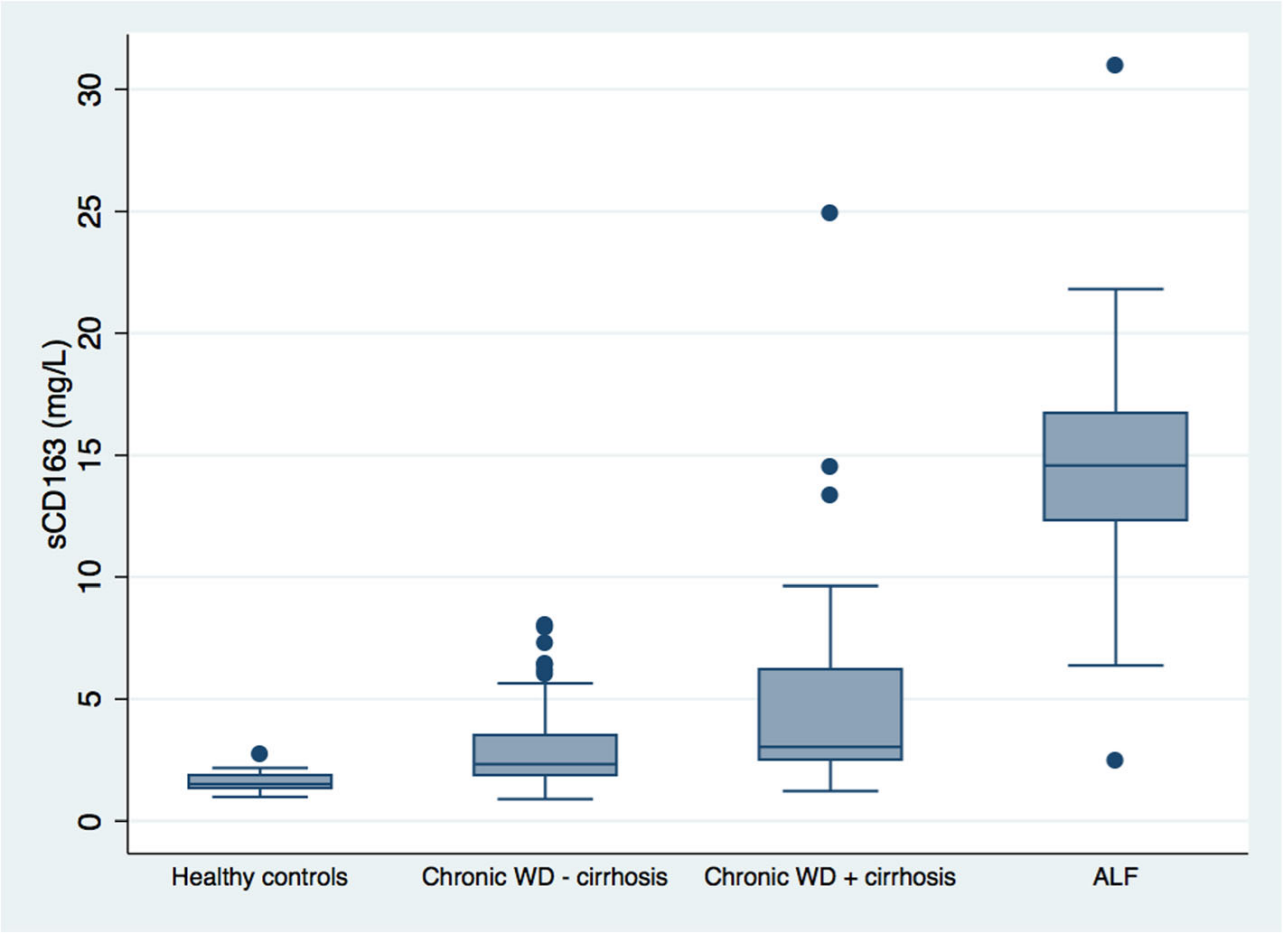
Soluble CD163 in healthy controls, chronic Wilson’s disease (WD) patients without or with cirrhosis at the time of diagnosis and in WD patients with acute liver failure (ALF). Boxes represent interquartile ranges with medians, whiskers show upper and lower adjacent values and closed circles outside values. *P* < 0.001 for all group comparisons

**Fig. 2:**
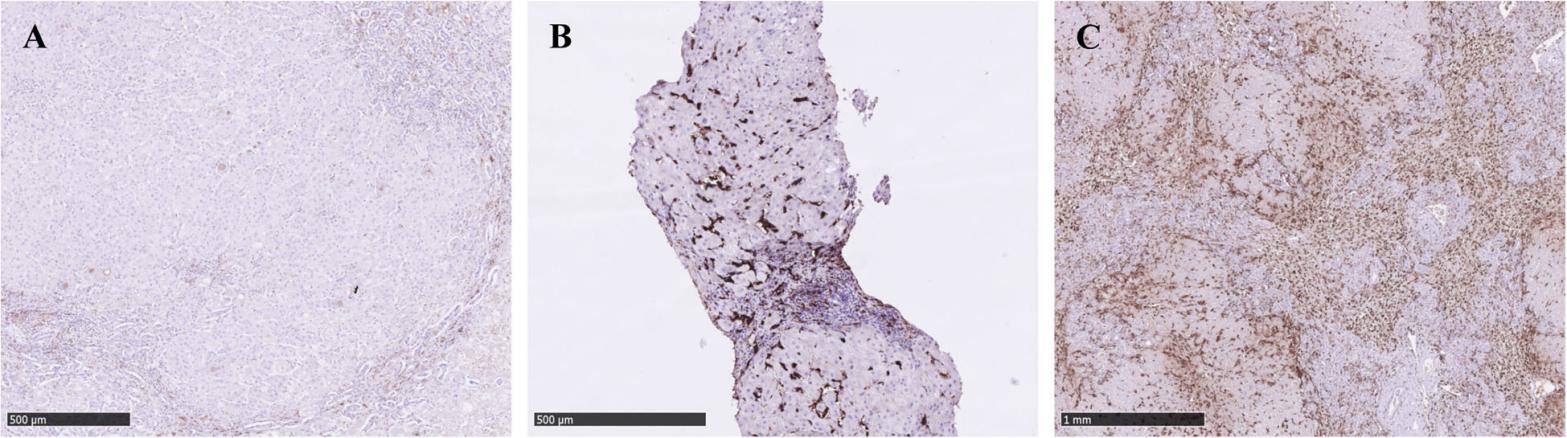
Immunohistochemical CD163 expression in liver tissue from Wilson’s disease patients with acute liver failure. Representative images of CD163 expression with semi-quantitative evaluation scores: (**a**) score 1; (**b**) score 2; and (**c**) score 3

In the US ALF cohort, baseline (i.e. day 1, *n* = 18; day 2, *n* = 10) sCD163 was 10-fold higher than in healthy controls (14.6 (2.5–30.9) mg/L vs. 1.5 (1.0–2.7) mg/L, *p* < 0.001) (Fig. 1). Out of 28 patients in the ALF cohort, 21 (75%) including two ALI patients received a liver transplant within 21 days of enrolment and 6 patients (21%) died, two after transplantation. Three patients (11%) including two ALI patients were spontaneous survivors at day 21. There was no difference in sCD163 levels between the ALI and ALF patients (15.2 (10.0–15.5) mg/L vs. 14.2 (2.5–30.9) mg/L, *p* = 0.75). The sCD163 levels at baseline were similar between the spontaneous survivors and the patients who received a liver transplant or died (14.1 (10.0–15.0) mg/L vs. 15.3 (2.5–30.9) mg/L, *p* = 0.55). Among the spontaneous survivors, sCD163 did not significantly change between baseline and last available measurement between day 4–7 (data not shown). There was no correlation between sCD163 and ALT, AST, bilirubin, albumin, INR, ceruloplasmin or 24-h urinary copper excretion. Immunohistochemical CD163 expression was observed in the liver tissue from WD patients from the US ALF cohort (Fig. 2). The semiquantitative score was 2.5 [1–3], none were graded 0, and the estimated area fraction of CD163 positivity was 4.5 (0.4–12.5).

### CD163 in chronic WD patients

In the German chronic cohort, sCD163 was almost 2-fold higher than in healthy controls (2.6 (0.9–24.9) mg/L, *p* < 0.001) but significantly lower than in ALF patients (*p <* 0.001) (Fig. 1). There was no difference in sCD163 levels according to subgroups based on initial presentation (asymptomatic, neurologic, hepatic, or mixed), but patients with cirrhosis at the time of diagnosis had higher sCD163 levels compared with patients without cirrhosis (3.0 (1.2–24.9) mg/L vs. 2.3 (0.9–8.0) mg/L, *p* < 0.001) (Fig. 1). Patients with cirrhosis had lower levels of albumin (*p* < 0.01), urea (*p* < 0.01) and WBC count (*p* < 0.05) and higher levels of INR (*p* < 0.001) compared with patients without cirrhosis. Further, patients with cirrhosis tended to be older (*p* = 0.06) and having higher levels of bilirubin (*p =* 0.06) and lower levels of serum copper (*p* = 0.09) compared with patients without cirrhosis.

There was no difference in sCD163 levels between patients with or without the point mutation H1069Q in one allele or both alleles (data not shown). Also, there was no difference between those with and without Kayser-Fleisher ring (data not shown).

**Fig. 3:**
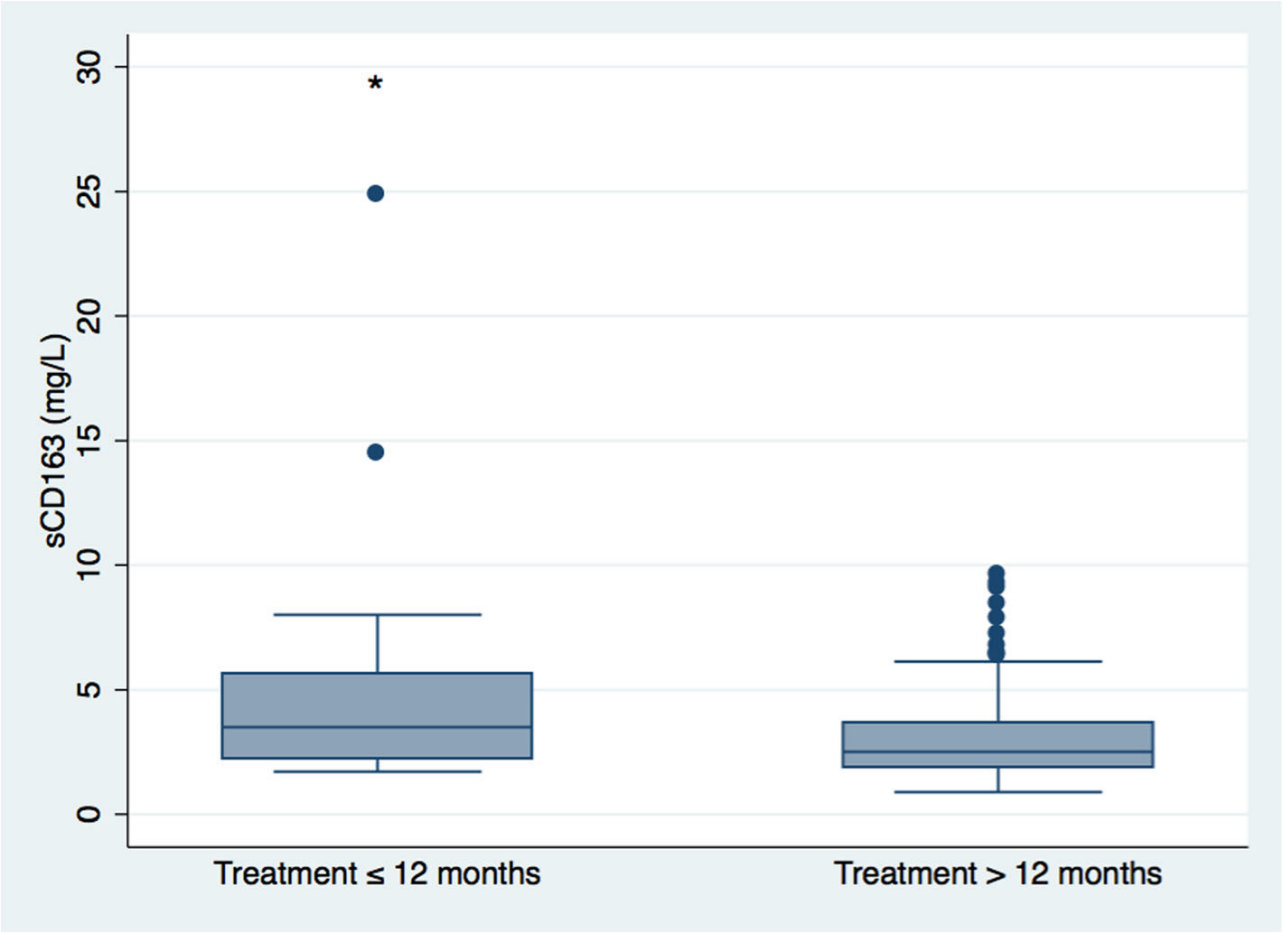
Soluble CD163 in chronic Wilson’s disease patients treated with current medical treatment for less than or equal to 12 months or more than 12 months. Boxes represent interquartile ranges with medians, whiskers show upper and lower adjacent values and closed circles outside values. * *P* = 0.02 treatment less than or equal to 12 months vs. treatment more than 12 months

**Fig. 4:**
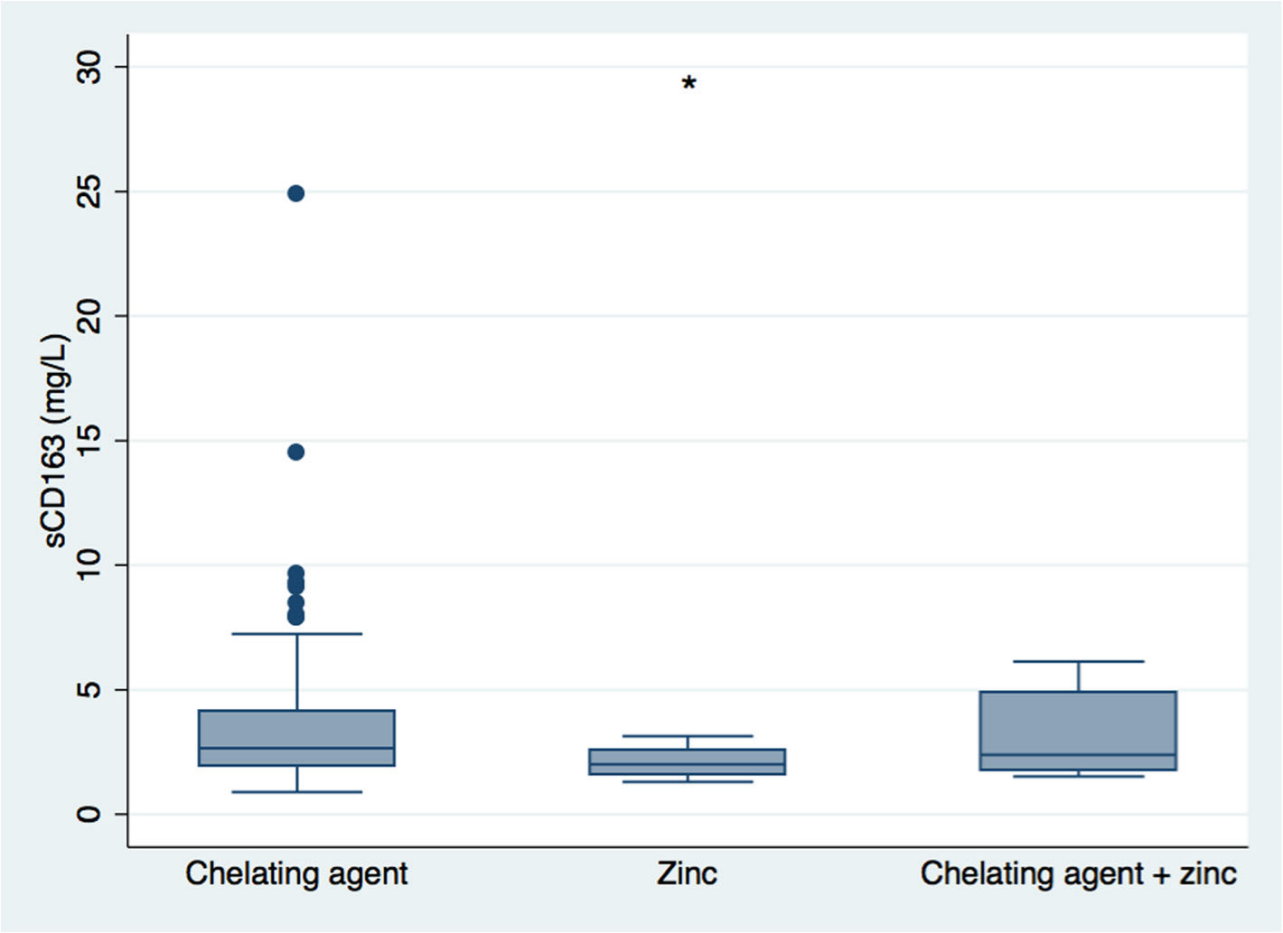
Soluble CD163 in chronic Wilson’s disease patients treated with a chelating agent, zinc or both. Boxes represent interquartile ranges with medians, whiskers show upper and lower adjacent values and closed circles outside values. * *P* < 0.001 zinc vs. chelating agent. There were no other differences between groups

Patients on current medical treatment for WD for more than 12 months had lower sCD163 levels compared with those treated for less than 12 months (2.5 (0.9–9.6) mg/L vs. 3.5 (1.7–24.9) mg/L, *p* = 0.02) (Fig. 3). Also, those with longer treatment duration were older and had lower levels of ALT, gamma-glutamyltransferase (GGT), and urinary copper (*p* < 0.05–0.001) (Supplementary Table 1). Regarding type of medication, i.e. a chelating agent, zinc or the combination, patients treated with zinc had lower sCD163 levels compared with those treated with a chelating agent (2.0 (1.3–3.1) mg/L vs. 2.7 (0.9–24.9) mg/L, *p* < 0.001), while there was no difference in sCD163 levels between the other treatment groups (Fig. 4). Further, there was no significant difference in treatment duration under the current medication between treatment groups (data not shown).

In the chronic cohort, sCD163 correlated positively with ALT (rho = 0.27), AST (rho = 0.53), GGT (rho = 0.48) and INR (rho = 0.41); and negatively with albumin (rho = − 0.37) (*p* ≤ 0.001, all). There was no correlation between sCD163 and bilirubin, ceruloplasmin or 24-h urinary copper excretion.

### Regression analysis for cirrhosis prediction using sCD163 in chronic WD patients

The multiple logistic regression analysis showed that sCD163 was not an independent predictor of cirrhosis. However, after a backward elimination procedure, sCD163, ALT and INR were independent variables in the final model (Supplementary Table 2) and included in a CD163-ALT-INR cirrhosis score:

CD163-ALT-INR = 0.91 * log sCD163 (mg/L) - 1.13 * log ALT (U/L) + 12.14 * log INR.

In non-parametric ROC analysis, sCD163 and the CD163-ALT-INR cirrhosis score predicted cirrhosis with an area under the ROC curve of 0.69 (95% confidence interval (CI): 0.59–0.79) and 0.80 (95% CI: 0.71–0.88), respectively (*p* = 0.02). The optimal cut-off value was at CD163-ALT-INR = − 2.19 (sensitivity = 69%, specificity = 81%, NPV = 87%, PPV = 59%).

## Discussion

This is the first study to investigate macrophage activation by sCD163 in a large sample of WD patients presenting with either ALF or chronic disease. Soluble CD163 was elevated in WD and with the highest levels in WD patients with ALF where immunohistochemical CD163-staining confirmed the presence of activated macrophages in the liver. In the chronic cohort sCD163 correlated with markers of liver injury and hepatocellular function and was higher in patients with cirrhosis than in those without cirrhosis at the time of diagnosis. Further, those with longer duration of treatment had lower sCD163 and improved liver function tests indicating better control of the liver disease. Our observations contribute to evidence that macrophage activation is important for the development of liver disease in WD and may suggest that sCD163 can be used as a marker of liver disease severity in WD patients.

The major strength of the present study is the large number of WD patients included from two different registries and the broad spectrum of liver disease phenotypes represented, ranging from the asymptomatic patient with only biochemical abnormalities to cirrhosis and also ALF. Study limitations include the use of a historical control group but sCD163 in that group (1.5 (1.0–2.7) mg/L) was similar to the normal values in the laboratory (1.7 mg/L (reference interval = 0.7–3.9 mg/L) based on 240 individuals [27]. Further, it is a limitation that time of initial diagnosis and blood sampling was separated in time. We therefore cannot be sure if the patients presenting with cirrhosis at diagnosis also had cirrhosis when the blood samples were taken. Finally, the major part of the study is cross-sectional which limits the causality aspects; however, we do provide follow-up measurements on sCD163 and data on day-21 mortality in patients from the US ALF cohort.

Compared with our previous study on sCD163 in ALF, sCD163 levels were lower (14.6 (2.5–30.9) mg/L) in our patients with WD and ALF than in patients with ALF due to acetaminophen, drugs, indeterminate or other unspecified reasons (median 21.1 mg/L) [22]. The sCD163 levels in the US cohort of patients with WD and ALF were similar to those previously presented in hospitalized patients with alcoholic hepatitis (median 15.4 mg/L) [19]. These data suggest massive activation of hepatic macrophages in the ALF situation, as also supported by the histological examination. The observed sCD163 levels in the patients from the German cohort with chronic WD were in agreement with our previous findings in a smaller pilot study [23]. In the large German cohort, sCD163 was not significantly different among clinical presentations: Asymptomatic, neurologic, hepatic, and mixed. There was no association between sCD163 and the presence of Kayser-Fleischer rings, the levels of serum ceruloplasmin or of urinary copper. Neither did we see an association with the H1069Q mutation and sCD163 but associations between genotype and clinical presentation are in general scarce in WD [28, 29]. At the same time, sCD163 levels were elevated in patients with cirrhosis independent of the clinical presentations. Our findings do not indicate that sCD163 is a marker for WD per se but instead suggest that macrophage activation and elevated sCD163 levels in WD reflect liver disease severity. This is in accordance with studies in other liver diseases [20, 30, 31].

Interestingly, patients pharmacologically treated for WD for less than 12 months had higher sCD163 levels than patients on long-term treatment suggesting a treatment effect with improved liver disease and reduced macrophage activation as observed in patients with chronic viral hepatitis [17, 32] or non-alcoholic fatty liver disease [33]. In addition, patients treated with zinc had lower sCD163 levels compared to chelating agents, which may suggest differences in treatment effects on macrophage activation. Of interest, CD163 is shed into the plasma on Toll-like receptor activation and parallels the response of tumour necrosis factor-α (TNF-α) to lipopolysaccharide (LPS) [27]. Zinc supplementation inhibits the LPS-induced hepatic release of TNF-α [34] and may in a similar way inhibit LPS-induced CD163 shedding partly explaining the lower sCD163 levels in zinc treated patients. However, no firm conclusions can be drawn based on our data and further studies are needed. The difference in sCD163 levels in treatment groups may also reflect selection bias since patients with elevated levels of ALT may change treatment from zinc to a chelating agent.

Soluble CD163 in the German chronic WD cohort correlated to biochemical markers of liver injury and hepatocellular function, while such correlations were not found in the US ALF cohort. This may indicate differences in the pathophysiological role for sCD163 in the two cohorts. In ALF due to WD ALT is only moderately elevated, and alkaline phosphatase surprisingly low [35], so these markers of liver injury develop differently in ALF due to WD than in other types of ALF. In accordance, in a larger sample of ALF patients of mixed origins sCD163 was in fact correlated to the liver function tests [22]. It may be speculated that in ALF patients, macrophage activation and sCD163 shedding may be caused by the massive hepatic necrosis with production of DAMPs. In contrast, in WD patients with chronic liver disease both DAMPs but also PAMPs including endotoxin and LPS from a leaky gut in patients with portal hypertension may be involved. Further, copper may itself be involved in macrophage activation and sCD163 shedding [36].

The severity of WD in the individual patient depends on the heterogenic expression of the range of symptoms such as neurological, psychiatric, hepatic, haemolysis and others. Most of these will not be reflected in sCD163. However, monitoring of liver disease progression and cirrhosis development in WD patients is a specific clinical challenge. As a single marker sCD163 predicted liver cirrhosis with modest accuracy with AUROC of 0.69. Using multiple logistic regression analysis with backward elimination the combination of sCD163, ALT and INR into a CD163-ALT-INR cirrhosis score resulted in a higher AUROC of 0.80. This score may represent a most needed tool for the non-invasive monitoring of disease progression and cirrhosis development in patients with WD but it needs validation in independent cohorts and with liver biopsy as gold standard.

## Conclusions

In conclusion, sCD163 was elevated in WD patients, especially in those with ALF. Further, sCD163 levels were higher in patients with cirrhosis compared to those without cirrhosis at the time of diagnosis, and associated with biochemical markers of liver injury and hepatocellular function. In patients with more than 1 year of treatment duration sCD163 levels were lower than in those with shorter duration. Thus macrophage activation is evident in WD and associates with the liver disease phenotype and biochemical parameters of liver disease. Our results indicate that sCD163 may reflect the liver disease severity in WD patients. Whether sCD163 could be a biomarker of progression of liver disease in WD should be examined and validated in larger prospective liver biopsy verified cohorts.

## Supplementary information

**Additional file 1: Supplementary Figure 1.** Histology illustrating scoring algorithm. Control liver tissue with low, moderate and high density of CD163 positive cells. (A) low density = score 1; (B) moderate density = score 2; and (C) high density = score 3. **Supplementary Table 1.** Characteristics for chronic Wilson's disease patients treated with current medical treatment for less than or equal to 12 months or more than 12 months. **Supplementary Table 2.** Multiple logistic regression model with soluble CD163, alanine aminotransferase, bilirubin, albumin, international normalized ratio, creatinine, age and gender as the explanatory variables for cirrhosis in patients with chronic Wilson’s disease.

## Data Availability

The datasets used and/or analysed during the current study are available from the corresponding author on reasonable request.

## References

[1] Ala A, Walker AP, Ashkan K, Dooley JS, Schilsky ML (2007). Wilson’s disease. Lancet..

[2] Roberts EA, Schilsky ML (2008). Diagnosis and treatment of Wilson disease: an update. Hepatology (Baltimore, Md).

[3] Clinical Practice Guidelines EASL (2012). Wilson’s disease. J Hepatol.

[4] Ferenci P (2014). Whom and how to screen for Wilson disease. Expert Rev Gastroenterol Hepatol.

[5] Weiss KH, Gotthardt DN, Klemm D, Merle U, Ferenci-Foerster D, Schaefer M (2011). Zinc monotherapy is not as effective as chelating agents in treatment of Wilson disease. Gastroenterology.

[6] Iorio R, D'Ambrosi M, Marcellini M, Barbera C, Maggiore G, Zancan L (2004). Serum transaminases in children with Wilson's disease. J Pediatr Gastroenterol Nutr.

[7] Schilsky ML, Scheinberg IH, Sternlieb I (1991). Prognosis of Wilsonian chronic active hepatitis. Gastroenterology..

[8] Camarata MA, Ala A, Schilsky ML (2019). Zinc maintenance therapy for Wilson disease: a comparison between zinc acetate and alternative zinc preparations. Hepatol Commun.

[9] Wu F, Wang J, Pu C, Qiao L, Jiang C (2015). Wilson's disease: a comprehensive review of the molecular mechanisms. Int J Mol Sci.

[10] Johncilla M, Mitchell KA (2011). Pathology of the liver in copper overload. Semin Liver Dis.

[11] Kubes P, Mehal WZ (2012). Sterile inflammation in the liver. Gastroenterology..

[12] Steib CJ (2011). Kupffer cell activation and portal hypertension. Gut..

[13] Boltjes A, Movita D, Boonstra A, Woltman AM (2014). The role of Kupffer cells in hepatitis B and hepatitis C virus infections. J Hepatol.

[14] Baffy G (2009). Kupffer cells in non-alcoholic fatty liver disease: the emerging view. J Hepatol.

[15] Moller HJ, Hald K, Moestrup SK (2002). Characterization of an enzyme-linked immunosorbent assay for soluble CD163. Scand J Clin Lab Invest.

[16] Kazankov K, Barrera F, Moller HJ, Rosso C, Bugianesi E, David E (2016). The macrophage activation marker sCD163 is associated with morphological disease stages in patients with non-alcoholic fatty liver disease. Liver Int.

[17] Laursen TL, Wong GL, Kazankov K, Sandahl T, Moller HJ, Hamilton-Dutoit S, et al. Soluble CD163 and mannose receptor associate with chronic hepatitis B activity and fibrosis and decline with treatment. J Gastroenterol Hepatol. 2017. 10.1111/jgh.13849 28618015

[18] Gronbaek H, Kreutzfeldt M, Kazankov K, Jessen N, Sandahl T, Hamilton-Dutoit S (2016). Single-Centre experience of the macrophage activation marker soluble (s)CD163 - associations with disease activity and treatment response in patients with autoimmune hepatitis. Aliment Pharmacol Ther.

[19] Sandahl TD, Gronbaek H, Moller HJ, Stoy S, Thomsen KL, Dige AK, et al. Hepatic macrophage activation and the LPS pathway in patients with alcoholic hepatitis: a prospective cohort study. Am J Gastroenterol. 2014. 10.1038/ajg.2014.262 25155228

[20] Gronbaek H, Sandahl TD, Mortensen C, Vilstrup H, Moller HJ, Moller S (2012). Soluble CD163, a marker of Kupffer cell activation, is related to portal hypertension in patients with liver cirrhosis. Aliment Pharmacol Ther.

[21] Gronbaek H, Rodgaard-Hansen S, Aagaard NK, Arroyo V, Moestrup SK, Garcia E, et al. Macrophage activation markers predict mortality in patients with liver cirrhosis without or with acute-on-chronic liver failure (ACLF). J Hepatol. 2015. 10.1016/j.jhep.2015.11.021 26639396

[22] Moller HJ, Gronbaek H, Schiodt FV, Holland-Fischer P, Schilsky M, Munoz S (2007). Soluble CD163 from activated macrophages predicts mortality in acute liver failure. J Hepatol.

[23] Bjorklund J, Laursen TL, Sandahl TD, Moller HJ, Vilstrup H, Ott P (2018). High hepatic macrophage activation and low liver function in stable Wilson patients - a Danish cross-sectional study. Orphanet J Rare Dis.

[24] Pfeiffenberger J, Mogler C, Gotthardt DN, Schulze-Bergkamen H, Litwin T, Reuner U (2015). Hepatobiliary malignancies in Wilson disease. Liver Int.

[25] Weiss KH, Merle U, Schaefer M, Ferenci P, Fullekrug J, Stremmel W (2006). Copper toxicosis gene MURR1 is not changed in Wilson disease patients with normal blood ceruloplasmin levels. WJG.

[26] Weiss KH, Runz H, Noe B, Gotthardt DN, Merle U, Ferenci P (2010). Genetic analysis of BIRC4/XIAP as a putative modifier gene of Wilson disease. J Inherit Metab Dis.

[27] Moller HJ (2012). Soluble CD163. Scand J Clin Lab Invest.

[28] Riordan SM, Williams R (2001). The Wilson's disease gene and phenotypic diversity. J Hepatol.

[29] Ferenci P, Stremmel W, Czlonkowska A, Szalay F, Viveiros A, Stattermayer AF (2019). Age and Sex but Not ATP7B Genotype Effectively Influence the Clinical Phenotype of Wilson Disease. Hepatology (Baltimore, Md).

[30] Holland-Fischer P, Gronbaek H, Sandahl TD, Moestrup SK, Riggio O, Ridola L (2011). Kupffer cells are activated in cirrhotic portal hypertension and not normalised by TIPS. Gut..

[31] Nielsen MC, Hvidbjerg Gantzel R, Clària J, Trebicka J, Møller HJ, Grønbæk H. Macrophage Activation Markers, CD163 and CD206, in Acute-on-Chronic Liver Failure. Cells. 2020;9(5). 10.3390/cells9051175 PMC7290463 32397365

[32] Laursen TL, Siggaard CB, Kazankov K, Sandahl TD, Moller HJ, Tarp B, et al. Time-dependent improvement of liver inflammation, fibrosis and metabolic liver function after successful direct-acting antiviral therapy of chronic hepatitis C. J Viral Hepat. 2019. 10.1111/jvh.13204 31502741

[33] Kazankov K, Tordjman J, Moller HJ, Vilstrup H, Poitou C, Bedossa P (2015). Macrophage activation marker soluble CD163 and non-alcoholic fatty liver disease in morbidly obese patients undergoing bariatric surgery. J Gastroenterol Hepatol.

[34] Zhou Z, Wang L, Song Z, Saari JT, McClain CJ, Kang YJ (2004). Abrogation of nuclear factor-kappaB activation is involved in zinc inhibition of lipopolysaccharide-induced tumor necrosis factor-alpha production and liver injury. Am J Pathol.

[35] Korman JD, Volenberg I, Balko J, Webster J, Schiodt FV, Squires RH, Jr., et al. Screening for Wilson disease in acute liver failure: a comparison of currently available diagnostic tests. Hepatology (Baltimore, Md). 2008;48(4):1167–74. 10.1002/hep.22446 PMC4881751 18798336

[36] Videla LA, Fernandez V, Tapia G, Varela P (2003). Oxidative stress-mediated hepatotoxicity of iron and copper: role of Kupffer cells. Biometals.

