# Supplementary material for: The macrophage activation marker soluble CD163 is elevated and associated with liver disease phenotype in patients with Wilson’s disease
Source: Orphanet J Rare Dis. 2020 Jul 2;15:173. doi: 10.1186/s13023-020-01452-2 (PMC7331244; doi:10.1186/s13023-020-01452-2)
Supplement: Supplementary file 1 — Additional file 1: Supplementary Figure 1. Histology illustrating scoring algorithm. Control liver tissue with low, moderate and high density of CD163 positive cells. (A) low density = score 1; (B) moderate density = score 2; and (C) high density = score 3. Supplementary Table 1. Characteristics for chronic Wilson's disease patients treated with current medical treatment for less than or equal to 12 months or more than 12 months. Supplementary Table 2. Multiple logistic regression model with soluble CD163, alanine aminotransferase, bilirubin, albumin, international normalized ratio, creatinine, age and gender as the explanatory variables for cirrhosis in patients with chronic Wilson’s disease. [file 13023_2020_1452_MOESM1_ESM.docx]

**Supplementary Information**

**Supplementary Figure 1. Histology illustrating scoring algorithm.** Control liver tissue with low, moderate and high density of CD163 positive cells. (A) low density = score 1; (B) moderate density = score 2; and (C) high density = score 3.


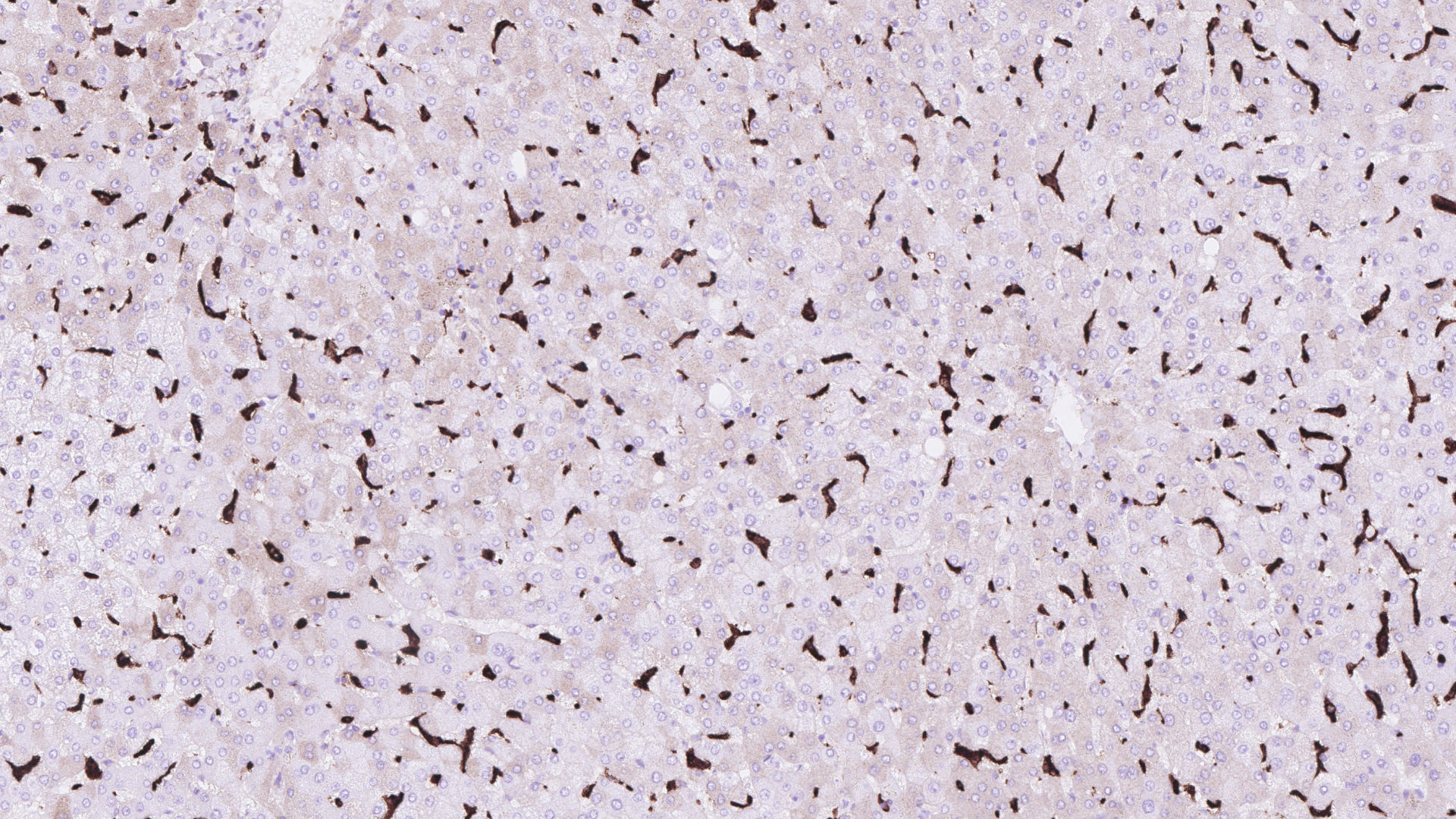


**A**


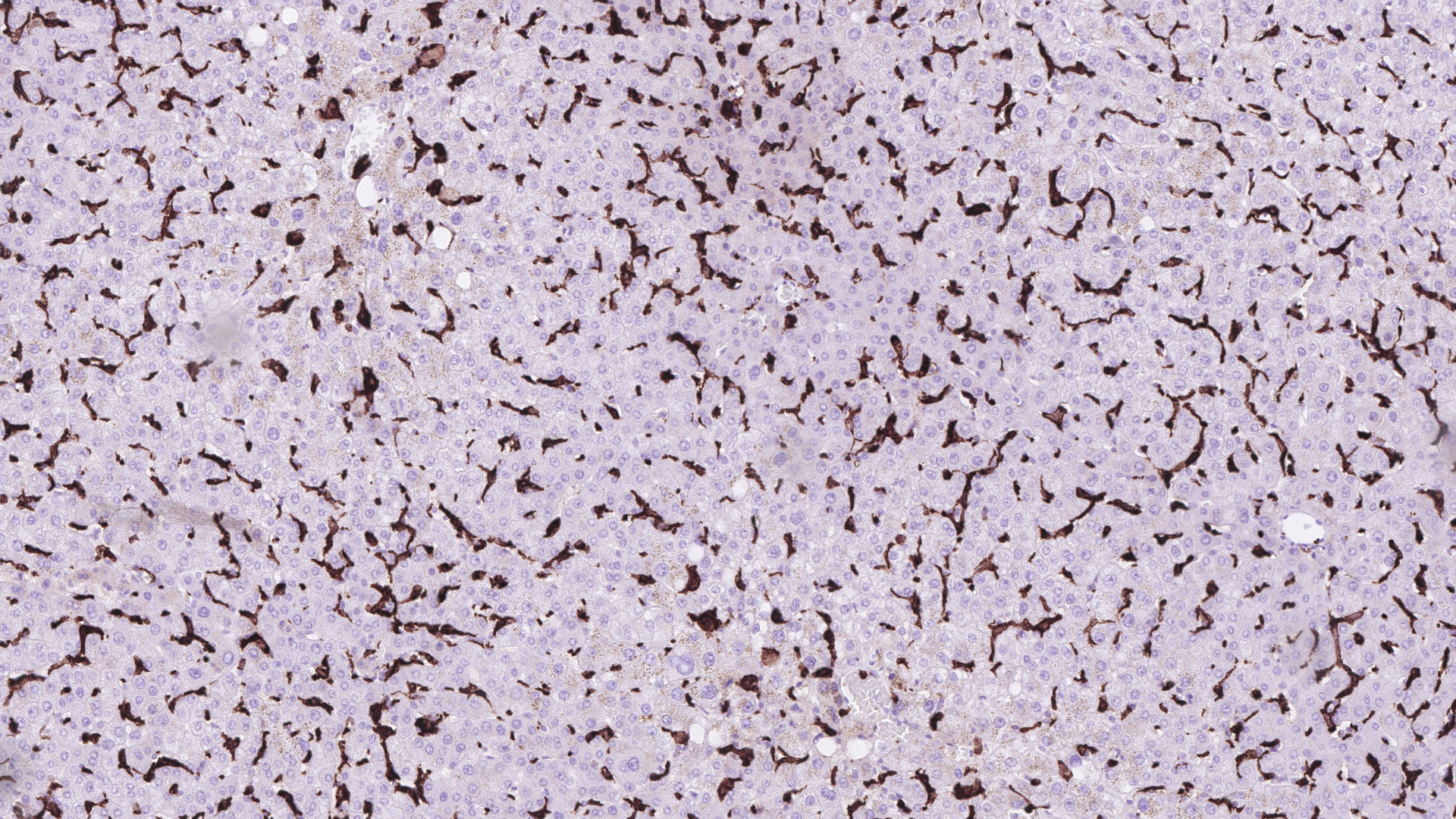


**B**


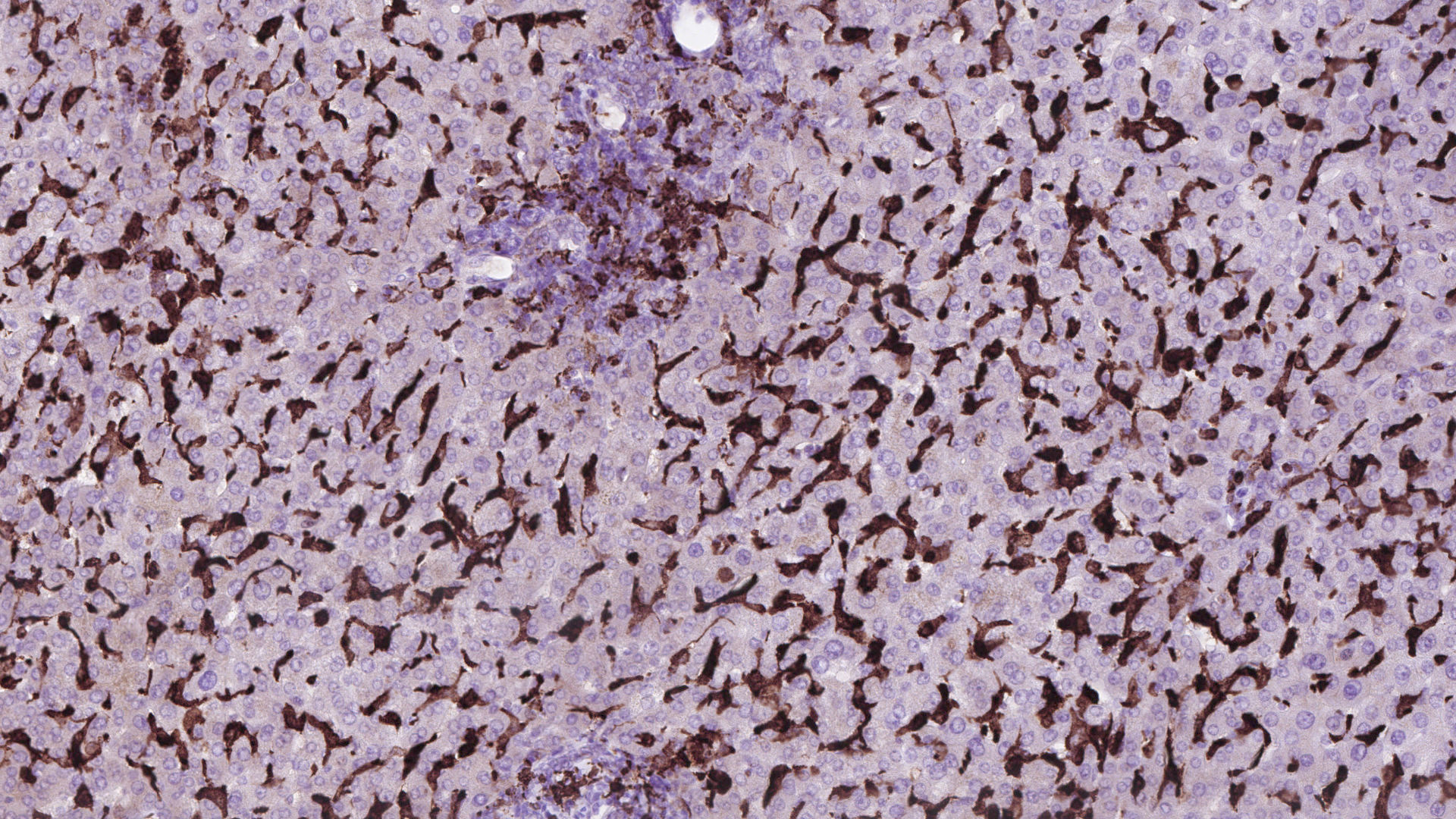


**C**

| **Supplementary Table 1.** | | |  |
| --- | --- | --- | --- |
|  | **Treatment ≤ 12 months**  **(n = 22)** | **Treatment > 12 months**  **(n = 123)** |  |
| Age (years) | 24 (18-52) | 38 (16-69)*** |  |
| Female gender | 12 (55%) | 66 (54%) |  |
| Cirrhosis at diagnosis | 6 (27%) | 33 (27%) |  |
| ALT (U/L) | 49 (20-436) | 34 (10-260)* |  |
| AST (U/L) | 30 (17-159) | 30 (8-580) |  |
| GGT (U/L) | 41 (11-398) | 31 (5-471)* |  |
| Bilirubin (mg/dL) | 0.6 (0.4-6.0) | 0.8 (0.3-7.8) |  |
| Albumin (g/L) | 44 (22-52) | 44 (29-53) |  |
| Urea (kU/L) | 5.7 (1.0-10.7) | 6.2 (2.4-11.9) |  |
| Creatinine (mg/dL) | 0.8 (0.4-1.0) | 0.7 (0.4-2.4) |  |
| WBC count (x10^9^/L) | 5.2 (2.8-12.2) | 5.9 (2.5-12.8) |  |
| INR | 1.1 (1.0-2.0) | 1.1 (0.9-1.3) |  |
|  |  |  |  |
| Serum copper (μmol/L) | 5.8 (0.8-29.9) | 4.3 (0.1-26.9) |  |
| Ceruloplasmin (g/L) | 0.10 (0.01-0.32) | 0.09 (0.01-0.28) |  |
| Urinary copper (μmol/day)^†^ | 5.4 (0.9-24.6) | 2.2 (0.2-27.5)** |  |
| ALT, alanine aminotransferase; AST, aspartate aminotransferase; GGT, gamma-glutamyltransferase; WBC, white blood cell; INR, international normalized ratio.  Data are medians (ranges) for continuous variables and total number (%) for categorical variables.  * p<0.05 compared with treatment ≤ 12 months  ** p<0.01 compared with treatment ≤ 12 months  *** p<0.001 compared with treatment ≤ 12 months  ^†^ n = 15 in treatment ≤ 12 months; n = 93 in treatment > 12 months | | |  |
|  | | |  |

**Supplementary Table 2. Multiple logistic regression model with soluble CD163, alanine aminotransferase, bilirubin, albumin, international normalized ratio, creatinine, age and gender as the explanatory variables for cirrhosis in patients with chronic Wilson’s disease.**

| **Independent variable** | **Regression coefficient** | **P-value** |
| --- | --- | --- |
| sCD163* | 0.71 | 0.19 |
| ALT* | -1.22 | 0.01 |
| Bilirubin* | -0.47 | 0.31 |
| Albumin* | -2.95 | 0.32 |
| INR* | 13.48 | 0.00 |
| Creatinine* | -0.32 | 0.81 |
| Age at blood sample collection* | 0.38 | 0.59 |
| Gender | 0.88 | 0.16 |

Regression coefficient and p-value for each independent variable are presented. The independent variables ALT and INR were statistically significant in the model. A backward elimination procedure resulted in sCD163, ALT and INR being significant in the final model (P≤0.05-0.001).

sCD163, soluble CD163; ALT, alanine aminotransferase; INR, international normalized ratio.

* Variable was logarithmically transformed.
